# Tetra­aquabis­(2-sulfamoylbenzoato)manganese(II)

**DOI:** 10.1107/S1600536808029450

**Published:** 2008-09-20

**Authors:** Rehana Akram, Waseeq Ahmad Siddiqui, M. Nawaz Tahir, Hamid Latif Siddiqui, Amjid Iqbal

**Affiliations:** a Department of Chemistry, University of Sargodha, Sargodha, Pakistan; b Department of Physics, University of Sargodha, Sargodha, Pakistan; c Institute of Chemistry, University of the Punjab, Lahore, Pakistan

## Abstract

In the title compound, [Mn(C_7_H_6_NO_4_S)_2_(H_2_O)_4_], the Mn atom, lying on an inversion center, exhibits a distorted octa­hedral coordination by six O atoms, two from carboxyl­ate groups and four from water mol­ecules. The SO_2_NH_2_ group is involved in a three dimensional polymeric hydrogen bonding network along with the water mol­ecules. π-Stacking inter­actions parallel to the *c* axis lead to a separation of 4.0050 (12) Å between the centroids of the benzene rings.

## Related literature

For related literature, see: Allen (2002 ▶); Aurengzeb *et al.* (1994 ▶); Eltayeb *et al.* (2008 ▶); Hulme *et al.* (1997 ▶); Siddiqui *et al.* (2007 ▶, 2008 ▶); Tahir *et al.* (1997 ▶); Zhang & Janiak (2001 ▶).
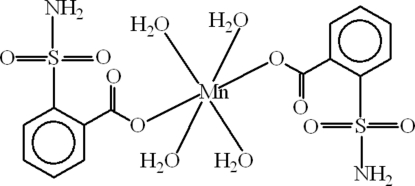


## Experimental

### 

#### Crystal data

[Mn(C_7_H_6_NO_4_S)_2_(H_2_O)_4_]
*M* *_r_* = 527.38
Monoclinic, 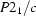

*a* = 15.2442 (4) Å
*b* = 8.2835 (2) Å
*c* = 7.9188 (2) Å
β = 99.971 (1)°
*V* = 984.85 (4) Å^3^
*Z* = 2
Mo *K*α radiation
μ = 0.95 mm^−1^
*T* = 296 (2) K
0.20 × 0.15 × 0.12 mm

#### Data collection

Bruker KAPPA APEXII CCD diffractometer
Absorption correction: multi-scan (*SADABS*; Bruker, 2007 ▶) *T*_min_ = 0.840, *T*_max_ = 0.895
10903 measured reflections
2445 independent reflections
2174 reflections with *I* > 2σ(*I*)
*R*_int_ = 0.027

#### Refinement

*R*[*F* ^2^ > 2σ(*F* ^2^)] = 0.029
*wR*(*F* ^2^) = 0.106
*S* = 1.05
2445 reflections
148 parameters
H atoms treated by a mixture of independent and constrained refinement
Δρ_max_ = 0.46 e Å^−3^
Δρ_min_ = −0.40 e Å^−3^

### 

Data collection: *APEX2* (Bruker, 2007 ▶); cell refinement: *APEX2*; data reduction: *SAINT* (Bruker, 2007 ▶); program(s) used to solve structure: *SHELXS97* (Sheldrick, 2008 ▶); program(s) used to refine structure: *SHELXL97* (Sheldrick, 2008 ▶); molecular graphics: *ORTEP-3 for Windows* (Farrugia, 1997 ▶) and *PLATON* (Spek, 2003 ▶); software used to prepare material for publication: *WinGX* (Farrugia, 1999 ▶) and *PLATON*.

## Supplementary Material

Crystal structure: contains datablocks global, I. DOI: 10.1107/S1600536808029450/fj2154sup1.cif

Structure factors: contains datablocks I. DOI: 10.1107/S1600536808029450/fj2154Isup2.hkl

Additional supplementary materials:  crystallographic information; 3D view; checkCIF report

## Figures and Tables

**Table:** 

Mn1—O1	2.1194 (13)
Mn1—O5	2.2582 (14)
Mn1—O6	2.1628 (14)
Mn1—O1^i^	2.1194 (13)
Mn1—O5^i^	2.2582 (14)
Mn1—O6^i^	2.1628 (14)
S1—O3	1.4313 (15)
S1—O4	1.4352 (16)
S1—N1	1.6223 (17)

**Table:** 

O1—Mn1—O5	93.95 (5)
O1—Mn1—O6	84.64 (5)
O1—Mn1—O5^i^	86.05 (5)
O1—Mn1—O6^i^	95.36 (5)
O5—Mn1—O6	84.27 (5)
Mn1—O1—C7	128.58 (11)

Symmetry code: (i) 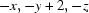.

**Table 2:** Hydrogen-bond geometry (Å, °)

*D*—H⋯*A*	*D*—H	H⋯*A*	*D*⋯*A*	*D*—H⋯*A*
O5—H5*A*⋯O3^ii^	0.86	2.02	2.869 (2)	167
O5—H5*B*⋯O2^iii^	0.97	1.83	2.775 (2)	164
O6—H6*A*⋯O5^iii^	0.94	1.85	2.765 (2)	164
O6—H6*B*⋯N1^iv^	0.86	2.25	3.008 (2)	148
N1—H11⋯O2	0.77 (3)	2.19 (3)	2.799 (2)	137 (2)
N1—H12⋯O4^v^	0.85 (2)	2.50 (2)	3.300 (2)	157 (2)
C3—H3⋯O4	0.93	2.40	2.834 (3)	108

Symmetry codes: (ii) 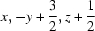; (iii) 
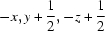
; (iv) 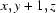; (v) 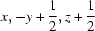.

**Fig. 1.**
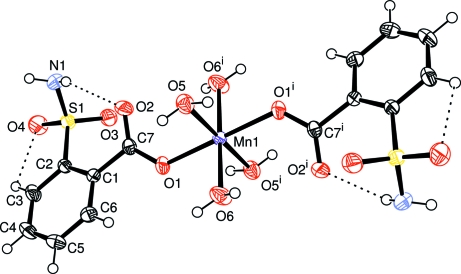
ORTEP drawing of the title compound, C14H20MnN2O12S2, with the atom numbering scheme. The thermal ellipsoids are drawn at the 50% probability level. H-atoms are shown by small circles of arbitrary radii. The intramolecular H-bonds are shown by dotted lines.

**Fig. 2.**
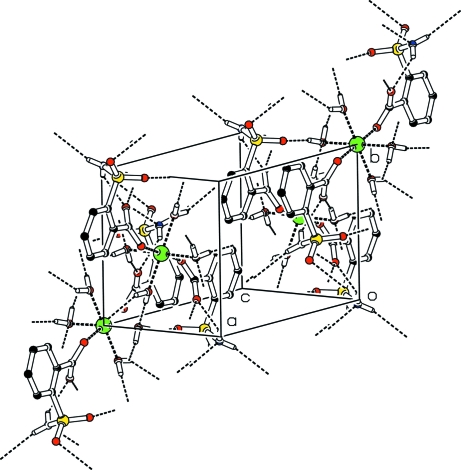
The packing figure (PLATON: Spek, 2003) which shows the three dimensional polymeric network through H-bonding. The H-atoms of benzene ring are not shown for clarity.

## supplementary crystallographic information

### Comment

The coordination chemistry of manganese in various oxidation states and in different combinations of donar environments like nitrogen and oxygen has been extensively investigated. The manganese complexes with Schiff base ligands have attracted considerable interest in the past decades and recently, due to their importance and variety of applications in chemistry, biology, physics and advanced materials (Eltayeb *et al.*, 2008). One class of high-valent manganese complexes receiving considerable attention is of those involving carboxylic acid and Schiff base ligands (Aurengzeb *et al.*, 1994; Hulme *et al.*, 1997; Zhang *et al.*, 2001). In continuation to the synthesis of benzene sulfonamide derivatives (Siddiqui *et al.*, 2008), we are also interested in the complexation of these ligands with human friendly transition metals. The title complex (I) is being reported in this context.

The CCDC search (Allen, 2002) showed that no crystal structure has been reported of manganese with sulfamoylbenzoate. The complexation of the Mn(II) with *o*-sulfamoylbenzoate confirmed that there is no coordination of SO_2_NH_2_ group. The Mn(II) is hexa-coordinated (Table 1) through carboxylate group (Fig 1) and four water molecules, whereas SO_2_NH_2_ is involved in the three dimensional network of H-bonding (Table 2). The Title compound (I) has shown a typical coordination geometry as is seen in most of the transition metal complexes (Tahir *et al.*, 1997) with monoanionic carboxylate ligands in the aqueous media. There exists a three dimensional polymeric network due to intra as well as intermolecular H-bonding. The molecules are further stabilized due to π-π-interaction at a distance of 4.0050 (12) Å, between the centroids of the benzene ring *Cg*···*Cg*^i^ [symmetry code: i = *x*, 3/2 - *y*, -1/2 + *z*].

### Experimental

A suspension of (1.0 g, 5.0 mmol) *o*-sulfamoyl benzoic acid (Siddiqui *et al.*, 2007), manganese acetate tetrahydrate (0.6 g, 2.5 mmol) and sodium carbonate (0.3 g, 2.5 mmol) was subjected to reflux in aqueous methanol (50%, 50 ml) for 4 h. The volume of the reaction mixture was reduced to half on rotary evaporator (11 torr) at room temperature and its pH was adjusted to 6 using hydrochloric acid (15%). The reaction mixture was then kept in ice-bath for 2 h. The off-white crystals were filtered, washed with cold distilled water and dried at room temperature. The product was recrystallized at 313 K from aqueous methanol to obtain colorless crystals.

m.p 498–503 K.

### Crystal data

**Table:**

[Mn(C_7_H_6_NO_4_S)_2_(H_2_O)_4_]	*F*(000) = 542
*M**_r_* = 527.38	*D*_x_ = 1.778 Mg m^−^^3^
Monoclinic, *P*2_1_/*c*	Mo *K*α radiation, λ = 0.71073 Å
Hall symbol: -P 2ybc	Cell parameters from 2445 reflections
*a* = 15.2442 (4) Å	θ = 1.4–28.3°
*b* = 8.2835 (2) Å	µ = 0.95 mm^−^^1^
*c* = 7.9188 (2) Å	*T* = 296 K
β = 99.971 (1)°	Prismatic, colourless
*V* = 984.85 (4) Å^3^	0.20 × 0.15 × 0.12 mm
*Z* = 2	

### Data collection

**Table:**

Bruker KAPPA APEXII CCD diffractometer	2445 independent reflections
Radiation source: fine-focus sealed tube	2174 reflections with *I* > 2σ(*I*)
graphite	*R*_int_ = 0.027
Detector resolution: 7.50 pixels mm^-1^	θ_max_ = 28.3°, θ_min_ = 1.4°
ω scans	*h* = −20→20
Absorption correction: multi-scan (SADABS; Bruker, 2007)	*k* = −11→7
*T*_min_ = 0.840, *T*_max_ = 0.895	*l* = −10→8
10903 measured reflections	

### Refinement

**Table:**

Refinement on *F*^2^	Primary atom site location: structure-invariant direct methods
Least-squares matrix: full	Secondary atom site location: difference Fourier map
*R*[*F*^2^ > 2σ(*F*^2^)] = 0.029	Hydrogen site location: inferred from neighbouring sites
*wR*(*F*^2^) = 0.106	H atoms treated by a mixture of independent and constrained refinement
*S* = 1.05	*w* = 1/[σ^2^(*F*_o_^2^) + (0.0727*P*)^2^ + 0.2877*P*] where *P* = (*F*_o_^2^ + 2*F*_c_^2^)/3
2445 reflections	(Δ/σ)_max_ < 0.001
148 parameters	Δρ_max_ = 0.46 e Å^−^^3^
0 restraints	Δρ_min_ = −0.40 e Å^−^^3^

### Special details

**Table:**

Geometry. Bond distances, angles *etc*. have been calculated using the rounded fractional coordinates. All su's are estimated from the variances of the (full) variance-covariance matrix. The cell e.s.d.'s are taken into account in the estimation of distances, angles and torsion angles
Refinement. Refinement of *F*^2^ against ALL reflections. The weighted *R*-factor *wR* and goodness of fit *S* are based on *F*^2^, conventional *R*-factors *R* are based on *F*, with *F* set to zero for negative *F*^2^. The threshold expression of *F*^2^ > σ(*F*^2^) is used only for calculating *R*-factors(gt) *etc*. and is not relevant to the choice of reflections for refinement. *R*-factors based on *F*^2^ are statistically about twice as large as those based on *F*, and *R*- factors based on ALL data will be even larger.

### Fractional atomic coordinates and isotropic or equivalent isotropic displacement parameters (Å^2^)

**Table:**

	*x*	*y*	*z*	*U*_iso_*/*U*_eq_	
Mn1	0.00000	1.00000	0.00000	0.0202 (1)	
S1	0.26720 (3)	0.44810 (6)	0.04336 (6)	0.0258 (1)	
O1	0.13332 (8)	0.92172 (17)	0.01231 (17)	0.0292 (4)	
O2	0.13202 (9)	0.68245 (19)	0.1401 (2)	0.0399 (4)	
O3	0.19410 (10)	0.46089 (19)	−0.09625 (18)	0.0352 (4)	
O4	0.33501 (10)	0.33230 (18)	0.0254 (2)	0.0415 (5)	
O5	0.00686 (10)	0.99300 (17)	0.28706 (18)	0.0334 (4)	
O6	0.05852 (9)	1.23701 (17)	0.0481 (2)	0.0411 (5)	
N1	0.22897 (12)	0.3966 (2)	0.2145 (2)	0.0324 (5)	
C1	0.26985 (10)	0.7841 (2)	0.0847 (2)	0.0213 (4)	
C2	0.31781 (10)	0.6410 (2)	0.0774 (2)	0.0226 (4)	
C3	0.41025 (11)	0.6438 (3)	0.0899 (3)	0.0330 (5)	
C4	0.45577 (12)	0.7888 (3)	0.1114 (3)	0.0407 (7)	
C5	0.40934 (13)	0.9313 (3)	0.1195 (3)	0.0363 (6)	
C6	0.31757 (12)	0.9290 (2)	0.1056 (2)	0.0275 (5)	
C7	0.16968 (10)	0.7939 (2)	0.0780 (2)	0.0224 (4)	
H3	0.44130	0.54792	0.08374	0.0395*	
H4	0.51741	0.79050	0.12051	0.0488*	
H5	0.43986	1.02886	0.13417	0.0436*	
H5A	0.06046	1.01646	0.33453	0.0400*	
H5B	−0.03444	1.07596	0.31053	0.0400*	
H6	0.28707	1.02566	0.11038	0.0330*	
H6A	0.02784	1.31074	0.10610	0.0493*	
H6B	0.11478	1.24615	0.08246	0.0493*	
H11	0.2043 (19)	0.467 (4)	0.248 (3)	0.0389*	
H12	0.2695 (16)	0.353 (3)	0.288 (3)	0.0389*	

### Atomic displacement parameters (Å^2^)

**Table:**

	*U*^11^	*U*^22^	*U*^33^	*U*^12^	*U*^13^	*U*^23^
Mn1	0.0192 (2)	0.0176 (2)	0.0239 (2)	0.0011 (1)	0.0040 (1)	0.0001 (1)
S1	0.0248 (2)	0.0198 (3)	0.0320 (2)	−0.0007 (2)	0.0028 (2)	−0.0006 (2)
O1	0.0223 (6)	0.0287 (7)	0.0359 (7)	0.0066 (5)	0.0028 (5)	0.0026 (5)
O2	0.0246 (6)	0.0393 (8)	0.0591 (9)	0.0024 (6)	0.0169 (6)	0.0163 (7)
O3	0.0339 (7)	0.0389 (8)	0.0303 (7)	−0.0078 (6)	−0.0011 (5)	−0.0005 (6)
O4	0.0396 (8)	0.0245 (7)	0.0612 (10)	0.0071 (6)	0.0114 (7)	−0.0040 (7)
O5	0.0280 (7)	0.0441 (9)	0.0276 (7)	0.0002 (5)	0.0035 (5)	−0.0005 (5)
O6	0.0301 (7)	0.0257 (7)	0.0684 (10)	−0.0049 (6)	0.0112 (6)	−0.0081 (7)
N1	0.0351 (9)	0.0273 (9)	0.0343 (8)	−0.0006 (7)	0.0046 (7)	0.0067 (7)
C1	0.0197 (7)	0.0219 (8)	0.0224 (7)	0.0007 (6)	0.0037 (5)	0.0033 (6)
C2	0.0185 (7)	0.0216 (8)	0.0274 (8)	−0.0005 (6)	0.0030 (6)	0.0009 (6)
C3	0.0199 (8)	0.0326 (10)	0.0461 (10)	0.0038 (7)	0.0049 (7)	0.0028 (8)
C4	0.0189 (8)	0.0454 (13)	0.0571 (13)	−0.0060 (8)	0.0050 (8)	0.0049 (10)
C5	0.0304 (9)	0.0318 (11)	0.0460 (11)	−0.0121 (8)	0.0045 (8)	0.0016 (9)
C6	0.0287 (8)	0.0223 (9)	0.0313 (9)	−0.0013 (7)	0.0045 (7)	0.0011 (7)
C7	0.0195 (7)	0.0248 (8)	0.0232 (7)	0.0035 (6)	0.0042 (5)	−0.0023 (6)

### Geometric parameters (Å, °)

**Table:**

Mn1—O1	2.1194 (13)	O6—H6B	0.8600
Mn1—O5	2.2582 (14)	N1—H11	0.77 (3)
Mn1—O6	2.1628 (14)	N1—H12	0.85 (2)
Mn1—O1^i^	2.1194 (13)	C1—C7	1.521 (2)
Mn1—O5^i^	2.2582 (14)	C1—C2	1.399 (2)
Mn1—O6^i^	2.1628 (14)	C1—C6	1.398 (2)
S1—O3	1.4313 (15)	C2—C3	1.396 (2)
S1—O4	1.4352 (16)	C3—C4	1.383 (3)
S1—N1	1.6223 (17)	C4—C5	1.384 (3)
S1—C2	1.7746 (17)	C5—C6	1.384 (3)
O1—C7	1.265 (2)	C3—H3	0.9300
O2—C7	1.234 (2)	C4—H4	0.9300
O5—H5A	0.8600	C5—H5	0.9300
O5—H5B	0.9700	C6—H6	0.9300
O6—H6A	0.9400		
			
S1···O2	3.0246 (16)	O4···H6^ii^	2.7600
S1···H6B^ii^	2.9200	O4···H3	2.4000
O1···O5	3.202 (2)	O4···H4^xi^	2.8900
O1···O6	2.883 (2)	O5···H6A^v^	1.8500
O1···O6^i^	3.1663 (19)	O6···H5A	2.9100
O1···O5^i^	2.989 (2)	O6···H5A^ix^	2.6500
O1···O3^iii^	3.228 (2)	O6···H5B^ix^	2.6500
O1···O2^iv^	3.068 (2)	N1···O2	2.799 (2)
O2···O1^iii^	3.068 (2)	N1···O6^ii^	3.008 (2)
O2···N1	2.799 (2)	N1···H6B^ii^	2.2500
O2···O6^i^	3.099 (2)	C6···O4^viii^	3.420 (2)
O2···S1	3.0246 (16)	C6···O3^iii^	3.389 (2)
O2···O3	2.895 (2)	C7···O3^iii^	3.254 (2)
O2···O5^v^	2.775 (2)	C7···O3	3.135 (2)
O3···C7	3.135 (2)	C3···H5^xii^	3.0300
O3···O5^iv^	2.869 (2)	C6···H12^iv^	3.08 (2)
O3···O6^ii^	3.136 (2)	C7···H11	3.03 (3)
O3···C6^iv^	3.389 (2)	C7···H5B^v^	2.9900
O3···O1^iv^	3.228 (2)	H3···O4	2.4000
O3···C7^iv^	3.254 (2)	H3···H3^xi^	2.5300
O3···O2	2.895 (2)	H4···O4^xi^	2.8900
O4···C6^ii^	3.420 (2)	H5···C3^xiii^	3.0300
O5···O6^v^	2.765 (2)	H5A···H6A^v^	2.2700
O5···O1^i^	2.989 (2)	H5A···O3^iii^	2.0200
O5···O6	2.967 (2)	H5A···O6^vi^	2.6500
O5···O6^vi^	3.055 (2)	H5B···O2^vii^	1.8300
O5···O2^vii^	2.775 (2)	H5B···C7^vii^	2.9900
O5···O1	3.202 (2)	H5B···H6A^v^	2.2900
O5···O3^iii^	2.869 (2)	H5B···O6^vi^	2.6500
O6···O5^vii^	2.765 (2)	H5B···H6A^vi^	2.5500
O6···O3^viii^	3.136 (2)	H6···O1	2.4900
O6···O5	2.967 (2)	H6···O4^viii^	2.7600
O6···O1^i^	3.1663 (19)	H6A···O5^vii^	1.8500
O6···O2^i^	3.099 (2)	H6A···H5A^vii^	2.2700
O6···N1^viii^	3.008 (2)	H6A···H5B^vii^	2.2900
O6···O5^ix^	3.055 (2)	H6A···O2^i^	2.8500
O6···O1	2.883 (2)	H6A···H5B^ix^	2.5500
O1···H6	2.4900	H6B···S1^viii^	2.9200
O1···H6B	2.7700	H6B···O3^viii^	2.6900
O1···H11^iv^	2.68 (3)	H6B···N1^viii^	2.2500
O1···H5B^i^	2.7300	H6B···H11^viii^	2.5100
O2···H11	2.19 (3)	H11···O2	2.19 (3)
O2···H5B^v^	1.8300	H11···C7	3.03 (3)
O2···H6A^i^	2.8500	H11···H6B^ii^	2.5100
O3···H6B^ii^	2.6900	H11···O1^iii^	2.68 (3)
O3···H5A^iv^	2.0200	H12···O4^xiv^	2.50 (2)
O4···H12^x^	2.50 (2)	H12···C6^iii^	3.08 (2)
			
O1—Mn1—O5	93.95 (5)	Mn1—O6—H6A	117.00
O1—Mn1—O6	84.64 (5)	H6A—O6—H6B	110.00
O1—Mn1—O1^i^	180.00	S1—N1—H12	111.1 (16)
O1—Mn1—O5^i^	86.05 (5)	S1—N1—H11	111 (2)
O1—Mn1—O6^i^	95.36 (5)	H11—N1—H12	115 (2)
O5—Mn1—O6	84.27 (5)	C2—C1—C6	117.85 (15)
O1^i^—Mn1—O5	86.05 (5)	C2—C1—C7	124.92 (14)
O5—Mn1—O5^i^	180.00	C6—C1—C7	117.19 (15)
O5—Mn1—O6^i^	95.73 (5)	S1—C2—C1	123.55 (12)
O1^i^—Mn1—O6	95.36 (5)	S1—C2—C3	115.65 (14)
O5^i^—Mn1—O6	95.73 (5)	C1—C2—C3	120.76 (17)
O6—Mn1—O6^i^	180.00	C2—C3—C4	120.1 (2)
O1^i^—Mn1—O5^i^	93.95 (5)	C3—C4—C5	119.80 (18)
O1^i^—Mn1—O6^i^	84.64 (5)	C4—C5—C6	120.2 (2)
O5^i^—Mn1—O6^i^	84.27 (5)	C1—C6—C5	121.27 (17)
O3—S1—O4	116.79 (9)	O2—C7—C1	118.68 (15)
O3—S1—N1	108.69 (9)	O1—C7—O2	126.13 (15)
O3—S1—C2	108.09 (9)	O1—C7—C1	115.15 (14)
O4—S1—N1	106.03 (9)	C2—C3—H3	120.00
O4—S1—C2	108.37 (8)	C4—C3—H3	120.00
N1—S1—C2	108.63 (8)	C3—C4—H4	120.00
Mn1—O1—C7	128.58 (11)	C5—C4—H4	120.00
H5A—O5—H5B	111.00	C4—C5—H5	120.00
Mn1—O5—H5B	105.00	C6—C5—H5	120.00
Mn1—O5—H5A	108.00	C1—C6—H6	119.00
Mn1—O6—H6B	120.00	C5—C6—H6	119.00
			
O5—Mn1—O1—C7	57.48 (15)	C7—C1—C2—S1	4.8 (2)
O6—Mn1—O1—C7	141.35 (15)	C7—C1—C2—C3	−177.97 (17)
O5^i^—Mn1—O1—C7	−122.52 (15)	C2—C1—C6—C5	−0.3 (2)
O6^i^—Mn1—O1—C7	−38.65 (15)	C7—C1—C6—C5	177.57 (17)
O3—S1—C2—C1	46.54 (16)	C2—C1—C7—O1	−150.20 (16)
O3—S1—C2—C3	−130.87 (15)	C2—C1—C7—O2	32.0 (2)
O4—S1—C2—C1	174.00 (14)	C6—C1—C7—O1	32.1 (2)
O4—S1—C2—C3	−3.41 (17)	C6—C1—C7—O2	−145.67 (16)
N1—S1—C2—C1	−71.23 (16)	S1—C2—C3—C4	178.11 (17)
N1—S1—C2—C3	111.36 (16)	C1—C2—C3—C4	0.6 (3)
Mn1—O1—C7—O2	4.9 (3)	C2—C3—C4—C5	−0.4 (3)
Mn1—O1—C7—C1	−172.72 (10)	C3—C4—C5—C6	−0.2 (3)
C6—C1—C2—S1	−177.56 (12)	C4—C5—C6—C1	0.6 (3)
C6—C1—C2—C3	−0.3 (2)		

Symmetry codes: (i) −*x*, −*y*+2, −*z*; (ii) *x*, *y*−1, *z*; (iii) *x*, −*y*+3/2, *z*+1/2; (iv) *x*, −*y*+3/2, *z*−1/2; (v) −*x*, *y*−1/2, −*z*+1/2; (vi) *x*, −*y*+5/2, *z*+1/2; (vii) −*x*, *y*+1/2, −*z*+1/2; (viii) *x*, *y*+1, *z*; (ix) *x*, −*y*+5/2, *z*−1/2; (x) *x*, −*y*+1/2, *z*−1/2; (xi) −*x*+1, −*y*+1, −*z*; (xii) −*x*+1, *y*−1/2, −*z*+1/2; (xiii) −*x*+1, *y*+1/2, −*z*+1/2; (xiv) *x*, −*y*+1/2, *z*+1/2.

### Hydrogen-bond geometry (Å, °)

**Table:**

*D*—H···*A*	*D*—H	H···*A*	*D*···*A*	*D*—H···*A*
O5—H5A···O3^iii^	0.86	2.02	2.869 (2)	167
O5—H5B···O2^vii^	0.97	1.83	2.775 (2)	164
O6—H6A···O5^vii^	0.94	1.85	2.765 (2)	164
O6—H6B···N1^viii^	0.86	2.25	3.008 (2)	148
N1—H11···O2	0.77 (3)	2.19 (3)	2.799 (2)	137 (2)
N1—H12···O4^xiv^	0.85 (2)	2.50 (2)	3.300 (2)	157 (2)
C3—H3···O4	0.93	2.40	2.834 (3)	108

Symmetry codes: (iii) *x*, −*y*+3/2, *z*+1/2; (vii) −*x*, *y*+1/2, −*z*+1/2; (viii) *x*, *y*+1, *z*; (xiv) *x*, −*y*+1/2, *z*+1/2.
